# The Social Determinants of Health in Military Forces of Iran: A Qualitative Study

**DOI:** 10.1155/2015/524341

**Published:** 2015-08-24

**Authors:** Mohammadkarim Bahadori, Hormoz Sanaeinasab, Mostafa Ghanei, Ali Mehrabi Tavana, Ramin Ravangard, Mazyar Karamali

**Affiliations:** ^1^Health Management Research Centre, Baqiyatallah University of Medical Sciences, Tehran, Iran; ^2^School of Management and Medical Information Sciences, Shiraz University of Medical Sciences, Shiraz, Iran

## Abstract

Providing effective health interventions and achieving equity in health need to apply the community-based approaches such as social determinants of health. In the military organizations, these determinants have received less attention from the military health researchers and policymakers. Therefore, this study aimed to identify and explain the social determinants affecting the health of military forces in Iran. This was a qualitative study which was conducted in 2014. The required data were collected through semistructured interviews and analyzed through Conventional Content Analysis. The studied sample consisted of 22 military health experts, policymakers, and senior managers selected using purposeful sampling method with maximum variation sampling. MAXQDA.2007 was used to analyze the collected data. After analyzing the collected data, two main contents, that is, “general social determinants of health” and “military social determinants of health,” with 22 themes and 90 subthemes were identified as the social determinants of military forces' health. Main themes were religious rule, spirituality promotion policies, international military factors, military command, and so forth. Given the role and importance of social factors determining the military forces' health, it can be recommended that the military organizations should pay more attention to these determinants in making policies and creating social, economic, and cultural structures for their forces.

## 1. Introduction

The social conditions in which people live have a considerable impact on their health. Situations such as poverty, poor nutrition, inadequate housing, unemployment, uncertain income, low education, social discrimination, culture, and access to healthcare services are the most important determinants of health and health inequalities. These factors, which affect the health and disease status of the population, are called social determinants of health [1, 2]. The factors and social conditions, in themselves or through their effects on each other, strongly affect health and can cause inequality and inequity in the individuals' health status among countries and within countries [3]. These factors are related to the physical, social, economic, and cultural environments in which people live and have effects on health through people's living conditions and their quality of life [2–5]. The results of experimental studies have shown that factors such as living environment, nutrition, education, social class, income, job, and employment influence health status and even their effects are much greater than those of healthcare [6–9].

Marmot and Wilkinson (2005) believe that the individuals' economic and social inequalities can lead to inequality in health [10]. Inequality in health can cause inequalities in the ability of individuals and their good performance and such inequalities can systematically result in the unequal social status and living conditions and, consequently, the failure of government social interventions [11]. Interventions in the area of social determinants of health should be dealt with, with a wide range of health determinants at all levels by the governments [12]. The health systems require attention to the determinants of health, which are the main causes of health inequalities, in order to achieve health equity [13]. The results of some studies show the fact that health intervention programs performed regularly are unable to decrease the inequalities in mortality between poor and rich people [14].

From the 1960s onwards, many activities and initiatives have been done to reduce the inequalities and inequities in health. The Great Britain plan has been to reduce health inequalities based on social determinants. The comprehensive public health policy in Sweden has been developed to create conditions for collective cooperation in order to achieve fair and equitable optimal health for all people. In Australia, the community empowerment program for prevention has been designed based on social determinants of health. The European policies have been dealing with the social classes, reducing stress in society, paying attention to the physical and psychological health and the development of children in their first years of life, paying attention to the social outcasts, employment and working conditions and environments, social support, addiction, food and nutrition, and transportation. And in the developing countries in Latin America, East Mediterranean, Asia, and Africa, the approach of social origins of diseases has been emphasized [15–18].

Because the inequalities in living and working conditions are preventable, trying to understand the mechanisms of social determinants effects on the individuals' health has been emphasized in order to eliminate the health inequalities in the world. Jong-Wook (2005) argued that the health interventions of countries aimed at fighting diseases and saving lives have been defeated and failed to reduce the inequalities and inequities [19, 20]. However, the World Health Organization (WHO) set up the Commission on Social Determinants of Health (CSDH) in 2005 [8]. One of the CSDH goals is to fill the gap in the scientific evidence about the social determinants and to develop and implement effective policies and procedures for eliminating health inequalities and inequities [7, 11, 21, 22]. The CSDH provided a conceptual framework for the action of member states representing the relationships between social determinants and their effects on the health in 2008 which was finalized in 2010 [12].

According to the conceptual framework, the following determinants or key factors affect health:

1. Social, economic, and political contextual factors, including governance, political institutions and economic processes, culture, and social system performance.
2. Structural factors, including education, income, gender, ethnicity, and employment status, which lead to the creation of social and economic inequalities and ultimately constitute a person's social class.
3. The middle part or intermediary factors indicating that the structural factors do not affect health directly. They influence health by intermediary factors.

**Figure 1:**
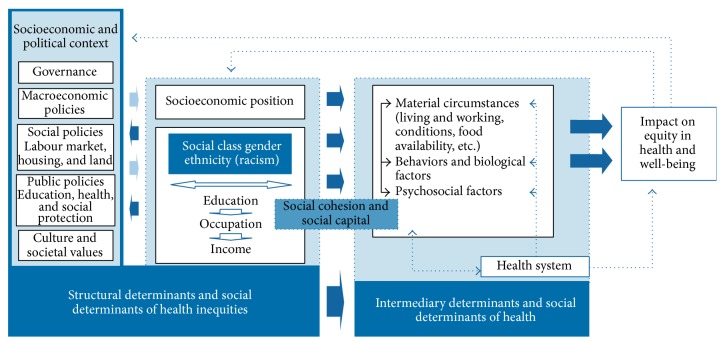
Final form of the CSDH conceptual framework [12].

These factors have effects on each other and ultimately on health (Figure 1) [23].

This framework provides guidance for policymakers about preventing health inequalities and inequities through acting on social determinants of health.

Strengthening national and global health equity means going beyond the current focus on primary and direct causes of diseases. The CSDH emphasizes the “causes of the causes” more than any other endeavor in the field of global health [4, 18, 22]. Under this approach to the concept of health, interventions and factors related to health are out of the scope of the individual behavior and specialized healthcare and have been developed into the wide range of individuals' socioeconomic life, including living and working environment, social protection, and education and training [23].

Iran, after its revolution and due to its significant improvements in the health sector especially in promoting social and health equity, has been considered by the World Health Organization as a partner country in choosing the social determinants of health approach in order to reduce health inequities. Despite the improvements in the health status and indicators such as decreased mortality and increased life expectancy at the national level in the past 30 years, the disparities and inequalities particularly between urban and rural areas are quite evident [24, 25]. The results of several studies carried out in Iran have indicated the effects of social determinants on the individuals' health [26–28]. Aghapoor and Mesri (2012) in a study showed that income inequality had a negative impact on the health indicators in Iran and the improvements in income distribution would raise the indicators of health [29]. Bahadori and Ravangard (2013) in their study have introduced factors such as the improvement of socioeconomic living conditions, paying attention to the early years of life, the improvement of the education quality, and the decreases in the unemployment and stress as the most important factors affecting health and providing the basis for improving equity in Iran [30]. The results of Hassanzadeh and colleagues' study (2013) indicated that the prerequisites for health equity were the social factors and the alimentation of inequalities in the social classes [31]. The results of Zaboli and colleagues' study (2014) confirmed the suitability of using the conceptual framework of social determinants of health, developed by WHO, for investigating the social determinants and health inequalities in Iran [32]. Most of the recent studies have been conducted on the effects of social determinants of health based on the WHO framework. Zaboli and colleagues in another study (2014) developed and expanded the WHO conceptual framework of social determinants of health and added other determinants such as the religious principles and faith, disaster and crisis, social and demographic factors, and international factor to it [33].

Military forces are considered as a population with specific characteristics whose health has always been emphasized in different countries [34].

Military forces are at risk of various diseases because of their collective life, missions away from home, and risky behavior. Determinants of health in the military forces include individual and psychosocial factors [35].

The conditions of being a military force and being employed in the armed forces, participation in the wars and critical missions, immigration, fear of loss and death, work-family and family-work conflicts, being away from family, and the lack of personal freedom are some of the psychosocial factors influencing the health of military personnel and their families.

Military organizations have personnel with different social and occupational positions, different needs and demands, and different behavior. Failure to respond to their needs and demands can cause their disappointment and disillusionment and unusual reactions [36].

Explaining and clarifying the views of the military health system experts in the field of social determinants of armed forces' health, including policymakers, managers, and researchers, is essential for better policymaking and planning in order to improve the military forces' health, and this happens through the use of a qualitative approach which explains the scientific and practical experiences of the participants in a study. On the other hand, despite the effects of social factors on the military forces' health, there are not enough studies in this field. Therefore, this study aimed to identify and explain the social determinants affecting the health of military forces in Iran.

## 2. Methods

This was a qualitative study which was conducted in 2014. The required data were collected through semistructured interviews and were analyzed through Conventional Content Analysis. Qualitative content analysis, as a research method, identifies the implicit and explicit themes in the text through subjective interpretation of the content of text data by a systematic classification process [37]. In the present study, the social determinants affecting the health of military personnel in Iran had been identified and explained using the comprehensive review of the military health system experts' views and their scientific and practical experiences.

### 2.1. Participants

The studied sample consisted of 22 military health experts, policymakers, and senior managers. This sample was selected using purposeful sampling method with maximum variation sampling [38]. The inclusion criteria for selecting the participants were as follows: formal employment in the military organization, working in the field of healthcare, having at least a master's degree, having at least 5 years of academic experience for experts, having at least 5 years of managerial experience, and having responsibility for military health policymaking and willingness to participate in the study and provide experiences.  Table 1 shows the demographic characteristics of participants in the study. Sampling continued until data saturation.

### 2.2. Collecting and Preparing Data

The required data were collected using the semistructured interview guide. This guide contained basic questions and was developed by the research team and its validity was confirmed by the experts and researchers in the field of military health. First, the research team prepared a list of experts according to the inclusion criteria with maximum variation diversity in terms of their job, academic and managerial experience, educational backgrounds, and expertise (because of the interdisciplinary nature of the study), and the researcher explained the aim of the study to the participants by visiting or calling them and provided them with the required information. After agreeing verbally to participate in the study, the researchers made an appointment with them. The interviews were conducted individually and in the appropriate places. The participants could leave the study at any time they wanted. The permission for recording the interviews was obtained from the participants.

Every interview began with a general question, “What are the social factors affecting the health of military forces?” And the participants were asked to express their opinions, experiences, and views. The next questions were asked based on the participants' experiences in order to achieve further information. Also, they were asked to give concrete examples, and some questions such as “What do you mean?,” “Would you please explain more about this?,” and “It is my understanding from your comments, is it correct?” were asked in order to increase the interviews depth and enrich the data.

The interviews were recorded using a digital voice recorder. Each interview lasted 37 to 94 minutes and took time according to the participants' tolerance level and interests. An interview session was held for each participant. The interviews continued until data saturation; that is, the new data entered into the study did not create new theme or change the existing themes [39]. In this study, the researchers carried out 20 interviews and reached data saturation. However, to ensure that there were not any new data, two other interviews were conducted. After each interview, it was transcribed immediately verbatim and word for word. For immersion in the data, which is necessary in the qualitative studies, the interviews transcription was reviewed several times.

### 2.3. Data Analysis

According to the Conventional Content Analysis, the analysis began simultaneously with data collection. Using this method, the codes and categories were extracted from the raw data directly and inductively. The codes and themes were identified through a systematic and transparent eight-step process. At first, the interviews were transcribed verbatim and typed immediately after each interview. To detect the semantic segments, each interview was read and reviewed. All words, sentences, and paragraphs that included the most important points about the social factors affecting the health of military personnel were determined as the semantic segments. Then, the semantic segments were reviewed several times and were coded based on the conceptual and semantic similarities. After extracting the original codes, similar codes were integrated and categorized based on the similarities. The categories and subcategories were compared with each other and the themes were determined by analyzing and interpreting these categories and subcategories [37, 40].  Table 2 illustrates the extracting codes, subcategories, and categories of a theme.

Based on the similarities between categories and subcategories, their development was continued, and, to ensure the data trustworthiness, the revision and comparison of categories with the data were performed. The themes were identified using careful and deep thinking, frequent reviews, and the comparison of categories with each other. MAXQDA.2007 was used to facilitate this process.

### 2.4. The Reliability and Validity of Results

During the study, four factors of Dependability, Credibility, Conformability, and Fittingness which have been provided by Lincoln and Guba, quoted from Shenton (2004) [41], were used to ensure the reliability and validity of data.

One of the best ways to increase the trustworthiness of data, which is the equivalent of validity and reliability in the quantitative studies, is the researchers' prolonged engagement with the study topic. Therefore, in the present study, the researchers had the prolonged engagement with the studied topic and context, and their relationships and contact with the participants helped to gain the participants' confidence and understand their experiences. The use of sampling methods with maximum variation can increase the Fittingness and transferability of results. Thus, the participants in the current study had various jobs and positions, academic and managerial experiences, and different expertise and job experiences in the field of military health.

The Credibility of data was enhanced through using member check, so that parts of the interviews with the related codes and categories emerging were given to the participants to review the results and verify them. The congruence of ideas derived from research data was compared under the participants' opinions. This was used to remove any ambiguity in coding and achieve the same concepts about the participants' ideas and statements. Also, the data saturation was used to increase the Credibility.

The Conformability of data was increased through audit trial, the impartiality in the interviews. Also, the researchers wrote and reported the research steps and processes accurately so that other researchers could conduct similar studies. In addition to the researchers, the results were reviewed and confirmed by two faculty members. The Dependability of data was enhanced through transcribing the interviews immediately and verbatim, using the external check and rereviewing the data. The Transferability was also increased through interviewing with different participants and providing their direct quotations and examples.

### 2.5. Ethical Considerations

In this study, obtaining informed consent, confidentiality of the participants' identity information, accurate and impartial transcription of the interviews, confidentiality of the participants' responses, the right to participate or leave the study, and confidentiality of the military information were considered as the ethical considerations.

## 3. Results

The process of qualitative content analysis for determining the social determinants of health in the military forces resulted in 275 initial codes. These codes, after several revisions and summarization based on the similarities, were categorized into 90 subconcepts (subcategories). The subconcepts were classified into 22 main concepts (categories). Categories and subcategories, based on their nature, were then categorized into two main themes: “general social determinants of health” and “military social determinants of health.” Table 3 illustrates the extracting theme, categories, and subcategories.

### 3.1. General Social Determinants of Health

#### 3.1.1. Physical Environment

One of the determinants of health in the military forces is physical environment and geographical conditions of working and living places which is for everyone. Some examples of quotes from experts in the study were as follows:

Environmental pollution is one of the social determinants affecting health. (E 7)

One of the social determinants is geographical location and climate conditions which impacts on the health and the characters are different in each environment. (E 13)

#### 3.1.2. Global Conditions

According to the country conditions, one of the determining factors raised by the experts was global issues affecting the health of military forces. One of the experts said the following:

… The resistive economic policies, dealing with sanctions and domineering countries, etc. are the social determinants affecting the health of both the public and military forces which get them into trouble mentally and psychologically. (E 4)

#### 3.1.3. Civil Social Determinants (Society)

The participants said the following in this regard:

We are an integral part of the environment and society. So, what is effective in the general conditions of the society is also effective in the military conditions. (E 10)

… Based on the WHO conceptual framework, all the context and structural determinants are effective in the armed forces and economic, social and cultural policies and other factors have great impacts on their health. (E 9)

The subthemes of the general social determinants of health in the armed forces have been shown in Table 3.

### 3.2. Military Social Determinants of Health

#### 3.2.1. The Sovereignty of God and Religion Rule

One of the important subthemes raised by the experts was the sovereignty of religion. According to the establishment of Islamic regulations and government in the country and task military forces to protect this system and, on the other hand, the existence of personal knowledge and beliefs based on the Islam principles and fundamentals, this factor was identified as a health determinant in the military forces. In this regard, the experts stated the following:

Because of the rich Islamic culture, we are far ahead of the World Health Organization and know the factors affecting health. (E 18)

One of the determinants of health in the military forces, which is more important than other determinants, is belief issues … A military person has difficult and harsh living conditions and needs to be prepared spiritually …  . When he is on a mission, he should make spiritually mental and psychological preparedness for himself and his family. (E 7)

The principles of religion sovereignty are for health improvement … Religious community starts with the health issues and considers cleanliness as a part of beliefs and trust in God, and believes that the health issues are very broad and widespread and makes them compulsory. (E 11)

#### 3.2.2. The Spirituality Promotion Policies

The spirituality promotion policies such as developing and implementing spirituality plans and establishing the system of values and competencies in order to strengthen spiritual, belief, and ideological dimension of military forces have significant effects on health. The experts expressed various views on this topic: good opportunity to inform the armed forces.

The improvement of military forces' health in the first step can be achieved by providing such training. However, the training and educational contents should be determined and provided. (E 7)

… If organizational policies and values are health-centered, they will have greater affecting the of military forces health … (E 8)

#### 3.2.3. Regional and International Military Factors

Given the role of military forces in the region and the international arena, the related issues such as the changes and security threats in the region, the patterns of interaction and confrontation with superpowers, and the diplomacy of military foreign relations were stated as the effective factors from the perspective of experts:

The changes and security threats in the region and the security problems have caused the alert conditions, insecurity and disturbance in the peace and comfort of the military personnel. (E 4)

If our policies on the foreign relations are confrontation with the world powers and in defense of the values, they will have effects on the lives and health of military forces. (E 16)

#### 3.2.4. Military Command and Management

Due to the hierarchical nature and the specific pattern of the military organization management, the military command and management was determined as one of the determinants affecting military forces' health. In this regard, the experts expressed the following:

The commander's personal characteristics, attitudes, culture, leadership and management style, the features of his command, and all issues relating to him affect the health of the armed forces. (E 20)

Policies and programs of the organization should be health-centered ones … To do anything, we should also pay attention to health. (E 8)

From the experts' perspective, the stewardship of armed forces' health is the commander's task. In this regard, one of the experts stated the following:

According to the Supreme Leader's order, the military forces' health and its improvement is one of the commanders' responsibilities. The healthcare system has the manager role and the commander is responsible for the armed forces' health. Paying attention to the armed forces' health is the inseparable and essential duty of a commander … (E 11)

#### 3.2.5. Military Defense and Security Policies

From the experts' perspective, the armed forces make general policies in several areas (Table 3) whose effects on the armed forces' health should be studied:

Policies made on the weapons and type of technologies, as well as the jobs which are defined such as Unmanned Aerial Vehicle (UAV) can affect the life and health of military forces. (E 16)

The policies of knowing the enemies and countering threats, organizational and hierarchical factors, environmental issues, barracks' environment, etc. are effective in the military forces' health. (E 17)

#### 3.2.6. Military Social Security Policies

According to the experts' views, a wide range of military social inequalities that cause inequalities in the military forces' health is affected by the social policies made to improve the livelihood and organizational support of the armed forces.

In this regard, the experts stated the following:

The military forces' policies affect other livelihood, welfare, and support policies … The type of decisions taken about the livelihood issues affect the health of the military forces. (E 3)

The military forces' welfare is one of the social factors affecting their health. (E 4)

Protecting the vulnerable groups, retirees and specific groups were introduced as an important way of eliminating health inequalities and developing social justice from the perspective of the experts:

At the end of the service and at the time of retirement, the responsibility of the armed forces has not ended. First, until the end of the service, their health should be protected through providing proper nutrition and adequate facilities for the armed forces. And then, at the time of retirement, they should receive health services appropriate to the elderly. (E 18)

The military person is healthy, but his child is sick. At this time, the military person is affected mentally and emotionally by his child's health. Therefore, to maintain the military forces' health, all social and psychological aspects threatening the person and his family's health should be paid attention. (E 7)

#### 3.2.7. Military Public and Supply Policies

If the policies of the military organization in the public and support fields are not health centered, they will negatively affect the military forces and their families' health and quality of life. In this regard, the experts expressed different views which have been shown in Table 3, some of which are as follows:

The nutrition of military forces is very effective in their health and is different from other people's nutrition. Because of the military forces' need for physical activities, their nutrition is different qualitatively and quantitatively. (E 7)

Personal equipment such as helmets, clothing, shoes and other requirements should be defined and have instructions for tasks and missions. According to the temperature, they need appropriate clothing and above all, they should be prepared for their tasks and missions and should have task fitness. (E 6)

In the field of engineering, if the patterns of housing, drinking water and other items are not health-centered, the problems will arise. (E 14)

#### 3.2.8. Culture and Values of the Military Organization

From the perspective of the experts, the organizational culture and values influence the health of the military personnel:

Each organization has a dominant culture which can improve or damage health. (E 8)

Because of the nature of military jobs, the personnel should more adhere to the principles, values and beliefs, and the spirituality should be given more importance. (E 1)

The military forces involve less interaction with the community that should be considered. (E 15)

#### 3.2.9. Military Sociodemographic Factors

The sociodemographic characteristics of the military personnel distinguish them from the grassroots and should be noted. The studied experts stated the following:

The military forces are known as a specific community and a sub-culture. (E 5)

In the military community, persons aged between 18 and 50 years and can have different health status. This means that with increasing the military forces' ages, their exposure to the environmental harmful and damaging factors will be increased … (E 20)

#### 3.2.10. Military Skills and Training

The rate of training and skills is one of the determinants of armed forces' health. The experts stated the following:

The armed forces' education levels and degrees lead to the creation of their socioeconomic classes which affect their job, income and knowledge. (E 1)

Providing theoretical and practical training, both initial training and in-service training, is a determinant of health and disease prevention; especially if the training is based on the person's mission and job requirements, for example, based on the mission and job requirements of someone who works in the Missile system. (E 6)

#### 3.2.11. Conditions of the Military Jobs

The experts agreed that one of the most important determinants of the military forces' health was their job conditions. In this regard, some subthemes such as the type and nature of job, military missions, type of military forces and organization, and job difficulties and limitations were identified (Tables 2 and 3).

#### 3.2.12. Organizational and Job Position

The social and organizational position, employee, clump, and field duty, the opportunities for organizational and job promotion, job security and satisfaction, salary, and livelihoods were the issues expressed by the experts:

In the Navy, the role of economic factors is highlighted and more important than other factors and affects the personnel's lifestyle and ultimately their health status. (E 9)

The socio-economic classes of military forces are important … According to the hierarchical system of the military forces, the equity and justice should be defined. (E 1)

#### 3.2.13. Social and Organizational Capital and Cohesion

The military forces' experts in the society and their social functions in the organization and society are the social health indicators. The studied experts stated the following:

The social capital is also a social determinant of military forces' health part of which is the armed forces' trust in their heads and authorities. (E 4)

The military personnel talk less about their positions in the society and this causes their social isolation. (E 5)

Some groups such as religious groups and boards, family groups or sports teams are a part of social environment … and, in fact, they with social relationship improve their members' health. (E 8)

#### 3.2.14. Social and Organizational Support

Strengthening the social and organizational support of the military forces has an important role in their health:

A military person as a combatant should have full health and fitness whose requirement is to have a support system. (E 3)

If we can provide the conditions under which people can share and reduce the mental and psychological tribulations, it will be helpful for their health and the negative impacts of psychosocial issues will become more tolerable. The social support begins from the family. (E 9)

Getting away from the family and the supportive roles of the organization and family can have effects on the military forces' health. (E 5)

#### 3.2.15. Amenities and Facilities of the Working Environment

One of the determinants of health is the facilities of the working environment. In this regard, the experts expressed the following:

To have a healthy working environment and control the harmful factors of the workplace, the personal protective equipment appropriate to the specific jobs should be developed and provided. (E 3)

Access to the sport and recreation facilities, as well as their development in the military barracks is one of the prevention factors in the field of SDH. (E 4)

#### 3.2.16. Conditions of the Military Forces' Living Environment

The studied experts emphasized that the conditions of the military forces and their families' living environment have great effects on their health:

The family should have good health and appropriate living facilities and welfare conditions. Livelihoods, welfare, housing, and healthy children are the factors in this sub-theme. (E 7)

The families of the military forces are away from the community and usually live in the military towns around the cities or away from the urban community and in an enclosed place, and have few interactions with the community. (E 13)

In this field, the effects of nutrition and housing on health have been proven. They affect the military forces and other people almost equally. (E 9)

#### 3.2.17. Biological and Psychological Factors

Biological, behavioral, and mental health factors are the factors that directly influence the health of the armed forces. Some examples of the views expressed by the experts are as follows:

Genetic backgrounds and the individuals' predispositions to suffering from diseases and injuries caused by the workplaces are effective in the military forces' health. (E 2)

According to the force structure and the military forces' jobs and missions, their types of diseases are different. For example, the navy and air forces suffer more from the job and occupational diseases. (E 14)

Military forces, compared with other people, are at risk of more occupational stresses, and more stressors are in the military environments than other jobs and professions environments. (E 1)

#### 3.2.18. Military Forces' Lifestyle

The experts considered lifestyle as the most important factor affecting the health of the military forces. Lifestyle includes nutrition, sports and physical fitness, healthy social relationships, high-risk behavior, belief and spiritual health, mental health, and occupational health. In this field, the experts stated the following:

Nutrition and dietary supplements are important for maintaining the military forces' health … In the barracks, proper diets are not followed and the dietary supplements required for the military forces are not paid full attention. Paying attention to the quantity and quality of the nutrition, micro-nutrients, vitamins, and macronutrients in the military forces is important. (E 6)

Physical fitness is an important factor in the armed forces' health. They should have a high level of physical fitness. They shouldn't be obese or overweight. Reduced physical activities, improper nutrition and social problems gradually affect the health of the military forces and provide conditions for developing diseases. (E 4)

A military person should show self-control which is based on his beliefs. (E 7)

#### 3.2.19. Military Health System

The military health system plays an important role in creating equality and equity in the military forces' health. Equitable access to the health services, military primary healthcare, interactions within the military health system, comprehensive monitoring of military forces' health, policymaking on and designing the interventions, and the structures and processes of the military health system are factors affecting the military forces' health [42].

In monitoring the health of the military forces, the strategies should move from Mass Screening to Screening. Currently, health monitoring system are based on the military forces' clump. Perhaps the monitoring of some diseases every four years is too early or too late. (E 16)

Having one-dimensional and above all, holistic treatment view in the field of military forces' health is harmful. (E 4)

## 4. Discussion

The approach of social determinants of health tries to address the inequities in the people's social status and their living and working conditions. Most inequalities can be avoided [32]. According to the conceptual framework of the CSDH, the identification of social determinants of health is “working beyond the first causes of diseases” [23, 43]. What can be inferred from the findings of similar studies in the field of social determinants of health [30, 32, 44, 45] is that the approach of social determinants of health in Iran is developing. Given the broadness of social determinants and their effects on health, the results of the present study showed that the health of Iranian military forces was affected by the globalization conditions, the economic, social, political, and cultural policies of the society, and the social factors of the military environment.

Based on the WHO conceptual framework, one of the context determinants is the macroeconomic policies which have effects on the inequalities in health through influencing other factors such as access to healthcare, healthy nutrition, education, housing, and transportation. The results of several studies, including Bambra and colleagues (2010), Pons-Vigués and colleagues (2014), and Scott and colleagues' (2012) studies, have confirmed the effects of public, economic, social, and cultural policies on health [46, 47]. The political and socioeconomic contexts, as structural determinants of inequalities in health, were in the CSDH conceptual framework, as well as the extended model of health inequalities in Iran [33] which confirms the results of the present study in military forces.

Health is a cross-sectoral issue and the growth of health indicators not only is affected by the health system performance, but needs to clarify and understand the role of cooperation, coordination, support, and interaction of all organizations, institutions, centers, and other parts outside the military health sector. The policies of the military forces towards the public support, social security, political security, and cultural and spiritual issues, which according to the results of the present study had effects on the health, indicate the broadness of factors affecting the military forces' health. The results of Aghapoor and Mesri's study (2012) on the effects of social and cultural factors on the soldiers' quality of life showed that their quality of life was affected by their religious orientation, the duration of the military service, self-esteem, age, education, and housing conditions [29]. The results of many studies conducted on the civilians' SDH have proven the effects of economic, social, and public policies, as well as the culture and social values on health [25, 30, 32, 48], which are consistent with the results of the present study.

Also, the results of several studies have approved the role of religion and spirituality in the health promotion. The results of Ahmadizadeh and colleagues' study (2014) showed that the relationship with God, providence and circumspection, and Unitarianism in the military forces had a very good status and their spiritual intelligence had a positive correlation with age. The results of Azad-Marzabadi and colleagues' study (2013) indicated that spirituality and spiritual intelligence were one of the most important factors affecting the employees' stress in a military university. The results of Mahboubi and colleagues' study (2012) showed that strengthening the spiritual health had effects on the physical, mental, and social health of the chemical warfare victims and controlled their social anxiety [49–51]. The results of Zaboli and colleagues' study (2014) also indicated that the religion was an important determinant of health inequalities [33]. In the present study, the participants believed that religious rule was a key determinant of the military forces' health.

According to the results of the current study, the stewardship of military forces' health is the commander's task and the military healthcare system has the manager role based on the SDH approach. Damari and colleagues (2013) in their study have described the role of Iran Ministry of Health and Medical Education (MOHME) in improving social health in the areas of knowledge production, support attraction, leadership and coordination, and special services of the ministry. Social health progress will not be achieved without intersectoral collaboration. Improvement of existing situation is not under duties and responsibilities of MOHME, so the proposed direction including vision, strategic objectives, and interventions for social health should be implemented partially by MOHME; remaining parts required advocacy to be done by other sectors [25]. The results of Karaminia and colleagues' study (2010) showed that leadership style used in military forces is associated with organizational culture and commitment of forces that can be direct or inverse [52] which is similar to the current study results.

From the perspective of experts in the present study, the organizational and job status affected income and socioeconomic position of the military forces. According to the WHO conceptual framework of structural determinants, as well as the results of Sajjadi and colleagues (2013), Kassani and colleagues (2012), Beheshtian and colleagues (2013), Heidarian and colleagues (2011), and Babakhani and Raghfar's (2010) studies, economic deprivation is one of the most important social determinants of health in Iran which results in reduced health related quality of life [53–57]. In Sajjadi and colleagues' study (2013), the association between economic inequalities, income, and health has been proven completely [57]. In other words, the socioeconomic status of a military person affects his health through influencing his living conditions. Therefore, the results of the present study confirm those of the mentioned studies.

Furthermore, the results of the current study showed that the conditions of the military jobs and their related factors were one of the social determinants of health. The results of a study have shown that the working conditions have significant effects on the inequalities [58]. The results of Kachooei and Fathi-Ashtiani's study (2013) indicated the positive association between the armed forces' job satisfaction and their quality of life [59]. Therefore, conditions of the military jobs should be considered carefully.

The results of Abedi and Mazruee's study (2010) showed that the military forces' job satisfaction was affected by their demographic factors such as experience, education, employee, and field duty [60] which are similar to those of the present study. From the participants' view in the current study, military demographic and social factors could be considered as a determinant of health in the military forces. Therefore, age, diversity, cultures and subcultures, and gender, as the military sociodemographic factors, were introduced as the determinants of health in the military forces.

In many studies, the researchers have investigated the association between health, education, and health literacy and reported a positive association between them based on the social determinants of health [44, 46, 47, 61, 62] which is consistent with the results of the current study. Accordingly, the military skills and education had been identified as a determinant of health in the military forces.

Biological and psychological factors were the other determinants of health in the military forces from the studied participants' viewpoint. The military jobs, different missions especially war conditions, psychological warfare, and the enemy propaganda operations can increase the risk of mental diseases in the military forces and disrupt their activities and tasks. Therefore, awareness of the factors causing stress in the military forces in special circumstances is essential [63, 64]. The results of Hosseini and colleagues' study (2012) [63] are similar to those of the present study.

Many of the common risk factors for human health such as smoking and stress are even with a higher prevalence in the military population than in the civilian population [65]. Job stress, complicated missions, rigid regulations, risks of injury, disability, imprisonment, and even death are some of the issues whose risks are much higher in the military jobs [63]. Azad Marzabadi (2010) in his study concluded that demographic and environmental factors affected stress in the armed forces and determined the regional problems or those related to the geographical and climatic conditions as the main stressors in the military personnel. Also, Azad-Marzabadi and Gholami-Fesharaki (2011) in another study showed that some factors affecting the job stress of military forces were age and job experience, and job satisfaction had an impact on all dimensions of the job stress [64, 66], which confirm the results of the present study.

From the perspective of studied participants, lifestyle was of utmost importance and one of the social factors affecting the military forces' health. Based on the results of Kassani and colleagues' study (2012), lifestyle is rooted in the culture and social and economic conditions [56]. According to the CSDH framework, lifestyle, which is one of the intermediate determinants, affects human health in many ways. The World Health Organization believes that many risk factors which are among the most common causes of death can be dealt with through lifestyle modifications. Therefore, one of the goals of the World Health Organization by 2020 is to promote the healthy lifestyle in the populations [67, 68]. The results of Feyzi and colleagues' study on the military personnel (2013) indicated a high prevalence of overweight, obesity and high body mass index, poor nutrition, and the lack of physical activities in the military personnel and emphasized the need for training to improve their lifestyle [69]. Tavakoli and colleagues in their studies (2011 and 2012) concluded that some factors such as economic factors, inadequate knowledge of commanders and military personnel about the fundamentals of proper nutrition, the use of old worn-out equipment in the eating places, improper methods of food processing and cooking, the employees' poor dietary habits and eating behavior, and lack of attention to the employees' food and nutritional tastes and culture were the reasons for poor nutritional styles in the military forces [68, 70]. The findings of the studies mentioned above about the effects of lifestyle on the military forces' health are consistent with those of the current study.

In the present study, social and organizational capital and cohesion and social and organizational support were introduced as two determinants affecting the military forces' health. Scientists and researchers have paid much attention to the association between social relationships and health. Most of the sociologists believe that social isolation or less social cohesion can damage health and, more importantly, increase the risk of premature death [71]. Bahadori and colleagues in their study in a military organization (2013) showed that intellectual growth, organizational support, and organizational culture had effects on the human resources' productivity [72].

Keyes and Shapirro (2004) believe that health studies conducted using biological samples put the emphasis more on the individual dimension of health; however, people are living within the structures and social relations. In fact, the public health is related to mental, personal, and social health. In Keyes and Shapirro's model, social well-being is a multidimensional concept that includes social integration, social contribution, social actualization, social coherence, and social acceptance [73]. The social health is one of the most fundamental criteria of each society's social welfare. Because of the stressful conditions of military forces, if they do not have any social protection, they will be exposed to different diseases and mental health problems. Therefore, countries consider all dimensions of health, especially the social dimension and family supportive system, in order to provide and maintain their military forces' readiness. For example, America's Army has designed the Comprehensive Soldier and Family Fitness (CSF2) program for continuous evaluation of the military forces [74]. Given that health has various physical, psychological, social, and spiritual dimensions, paying attention to the social capital can improve the health dimensions of the military forces.

Investigating the different psychological aspects and the life quality of military forces is important for improving their military power and efficiency and therefore improving the military power [75]. Quality of Work Life (QWL) is a multifaceted and relative concept that is influenced by time, place, and personal and social values. Managers should pay more attention to their employees' QWL. They can create some QWL programs and open communication ways to influence their employees' participation in making decisions about their jobs and organizations. These programs need special consideration for the new employees and those who are in the lower levels of jobs [76]. Job satisfaction is one of the most challenging organizational concepts and the basis of many policies and guidelines of management for promoting the efficiency and productivity of organization [60]. Military forces are at risk of various diseases because of their collective life, missions away from home, and risky behavior [77]. Based on the results of the present study, the conditions of the military forces' jobs and living environment are important determinants affecting the military forces' health.

The present study had some limitations; the most important ones were the lack of studies in the field of military forces' health and the lack of scientific evidence for and studies on social determinants affecting the military forces' health.

**Figure 2:**
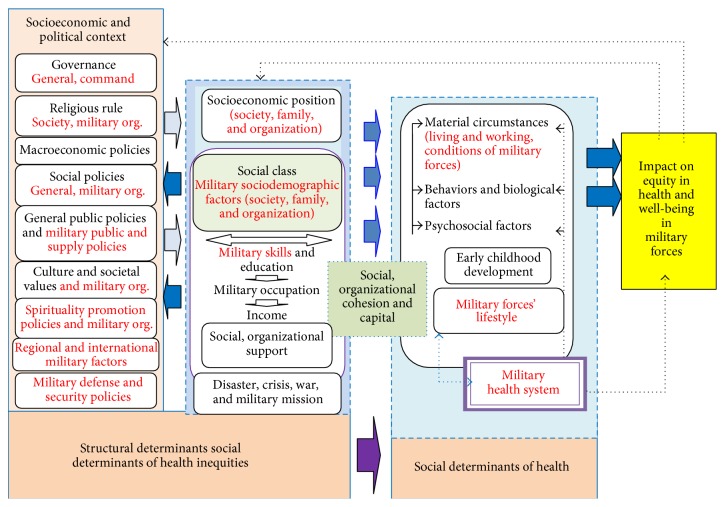
The extended conceptual framework of social determinants of military forces' health in Iran based on the conceptual framework of CSDH [12].

According to the results of the present study, general and military social determinants of health were factors affecting the military forces' health. In the present study, based on the conceptual framework of the World Health Organization and the experts' viewpoints, the framework of social determinants of health was reviewed and, according to the conditions of the military environments, the new themes and subthemes were identified and added to it. The related extended framework has been shown in Figure 2. The military organizations should pay more attention to these determinants in making policies and creating social, economic, and cultural structures, and all military decisions should be health oriented. It can be recommended to carry out studies on and gather evidence for this framework in order to clarify the effects of these determinants for commanders, managers, and policymakers.

## Figures and Tables

**Table 1:** The demographic characteristics of participants in the study (*n* = 22).

Variables		Frequency (%)
Age (years)	35–44	6 (27)
	45–54	12 (55)
	>55	4 (18)

Managerial experience (years)	5–10	5 (23)
	11–20	7 (32)
	>20	10 (45)

**Table 2:** The extracted categories, subcategories, and codes of a theme.

Themes	Categories	Subcategories	Codes	Semantic segments
Military social determinants of health	Conditions of the military jobs	Type and nature of the job	Line and staff and operational jobs	“The operational jobs have physical activity and mobility constantly. However, staff jobs are very sedentary and job tasks can be highly effective on the military forces' health” (P 20)
			Occupational corps/branches	“The equipment and the type of occupational corps/branches such as infantry, artillery, and so forth, are introduced as determinants” (P 15)
		Military missions	Types of missions	“The missions of military forces are different based on the conditions of wartime or peacetime” (P 16)
			The place and region of missions	“When a mission is given to a military person, the environmental and geopolitical issues such as humidity, temperature, wind, and so forth, are not being considered” (P 6)
		Type of military forces and organization	Employment in the ground forces, air forces, or navy	“According to the force structure and the military forces' jobs and missions, their types of diseases and health needs are different. Therefore, the force structure is a determinant” (P 14)
			The differences in the military and police forces	“Police forces are more interacting with people and have various stresses which can be transferred to their families” (P 4)
		Difficulties and limitations of the military jobs	Military rigid and inflexible regulations	“The military disciplinary regulations are the other social factors affecting health in the military forces. There are rigid and inflexible regulations and rules” (P 13)
			Getting away from family and society	“A person who is on a mission in a naval ship for six months gets away from his family and friends and the social environment, and can be predisposed to particular diseases” (P 8)

**Table 3:** Social determinants of health in the military forces from the perspective of studied experts.

Themes	Main concepts (categories)	Related subconcepts (subcategories)
General social determinants of health	Physical environment	Environmental pollution/geographical conditions
	Global conditions	International sanctions and domineering superpowers/global health policies
	Civil social determinants of the society (context, structural and intermediate factors)	Sociopolitical sovereignty and leadership/macroeconomic policies/social policies: labor, land, and housing/public policies of the government: education, health, transportation, agriculture, and so forth/culture, social values, and customs of the society/socioeconomic status of the military forces in the society/support and preventive institutions of the society/demographic and social factors/psychological factors in the society/events, crises, and disasters and accidents/early childhood periods/national health system

Military social determinants of health	The sovereignty of God and religious rule	God and monotheistic worldview/personal knowledge, beliefs, and doctrines
	The spirituality promotion policies	Spiritual promotion/system of values and competencies
	Regional and international military factors	Changes and security threats in the region/patterns of interaction and confrontation with superpowers/diplomacy of military foreign relations
	Military command and management	Stewardship of the military forces' health/commander's characteristics/the content of the policies and commands
	Military defense and security policies	technology and weapons policies/the doctrine of combat readiness and power/security and defense missions/knowing the enemies/professional military jobs
	Military social protection policies	Living improvement policies/insurance/housing/welfare services for the employees/specific and vulnerable groups
	Military public and supply policies	Logistics and nutrition/human resources management/training and empowerment/military health management/coordination and support/physical activity and fitness/engineering and construction
	Culture and values of the military organization	Organizational culture/military regulations/personal beliefs
	Military sociodemographic factors	Gender and racial factors/demographic variables (including age and experience)/culture and family roots (including ethnicity)
	Military skills and education	Initial training/in-service empowerment/health literacy/education
	Conditions of the military jobs	Type and nature of job/military missions/type of military forces and organization/job difficulties and limitations
	Organizational and job position	Social and organizational status/employee, clump, and field duty/opportunities for organizational and job promotion/job security and satisfaction/salary and livelihoods
	Social and organizational capital and cohesion	Personal relationships and organizational affiliation/cultural and regulatory institutions in the organization/social groups
	Social and organizational support	Supporting institutions in the organization/specific groups/family support institutions
	Amenities and facilities of the working environment	Individual requirements/health, sports, and recreation infrastructures/environment and tools safety
	Conditions of the military forces' living environment	Family health/life needs/access to services
	Biological and psychological factors	Individual constitutional actors/occupational injuries and pathogens/climate and ecological conditions/stress
	Military forces' lifestyle	nutrition/sports and physical fitness/healthy social relationships/high-risk behavior/belief and spiritual health/mental health/occupational health
	Military health system	Equitable access to the health services (geographical, technological, financial, etc.)/military primary health care/interactions within the military health system/comprehensive monitoring of military forces' health/policymaking on and designing the interventions/structures and processes of the military health system
